# Supplementary material for: Bacterial endosymbionts influence host sexuality and reveal reproductive genes of early divergent fungi
Source: Nat Commun. 2017 Nov 29;8:1843. doi: 10.1038/s41467-017-02052-8 (PMC5705715; doi:10.1038/s41467-017-02052-8)
Supplement: Supplementary file 2 — Supplementary Information [file 41467_2017_2052_MOESM2_ESM.pdf]

Supplementary Table 1. Spontaneous loss of endobacteria and asexual sporulation during serial propagation of *Rm* ATCC 52813 lines 1-1, 1-2, 2-1, 2-2, 3-1, 3-2 via single spores and mycelial fragments. P, present; A, absent.

Generation	Sporulation in single spore lines						Sporulation in mycelial fragment lines					
	1-1	1-2	2-1	2-2	3-1	3-2	1-1	1-2	2-1	2-2	3-1	3-2
1	P	P	P	P	P	P	P	P	P	P	P	P
2	P	P	P	P	P	P	P	P	P	P	P	P
3	P	P	P	P	P	P	P	P	P	P	P	P
4	P	P	P	P	P	P	P	P	P	P	P	P
5	**A**	**A**	P	P	P	P	P	P	P	P	P	P
6	**A**	**A**	P	P	P	P	P	P	P	P	P	P
7	**A**	**A**	P	P	P	P	P	P	P	P	P	P
8	**A**	**A**	P	P	P	P	P	P	P	P	P	P
9	**A**	**A**	P	P	P	P	P	P	P	P	P	P
10	**A**	**A**	P	P	P	P	P	P	P	P	P	P
11	**A**	**A**	P	P	P	P	P	P	P	P	P	P
12	**A**	**A**	P	P	P	P	P	P	P	P	P	P
13	**A**	**A**	P	P	P	P	P	P	**A**	P	P	P
14	**A**	**A**	P	P	P	P	P	P	**A**	P	P	P
15	**A**	**A**	P	P	P	P	P	P	**A**	P	P	P

Supplementary Table 2. Effects of various environmental factors on asexual and sexual reproduction in B(+) and B(–) isolates of *Rm* ATCC 52813 and ATCC 52814. +, normal reproduction; ++, exceptionally robust reproduction; ±, reduced reproduction; A, reproduction absent; X, failure to grow vegetatively; (Y), accumulation of β-carotene; (YY), rapid accumulation of β-carotene; NA, data not available. Each observation summarizes three replicate cultures.

Environmental factor	Wild type fungi harboring endobacteria				Fungi cured of endobacteria			
	ATCC 52813		ATCC 52814		ATCC 52813		ATCC 52814	
level	asexual sporulation	mating	asexual sporulation	mating	asexual sporulation	mating	asexual sporulation	mating
**Thiamine**	+	+	+	+	A	A	A	A
**β-carotene**	+	+	+	+	A	A	A	A
**$NaNO_3$**	+	+	+	+	A	A	A	A
**$NaNO_2$**	±	+	±	+	A	A	A	A
**Mated culture extract**	+	+	+	+	A	A	A	A
**Humidity**								
**high**	+	+	+	+	A	A	A	A
**low**	++	+	++	+	A(Y)	A	A(Y)	A
**Light**								
**ambient**	+	+	+	+	A	A	A	A
**darkness**	+	+	+	+	A	A	A	A
**Cold treatment**								
**0 weeks**	+	+	+	+	A	A	A	A
**1 week**	+	+	+	+	A	A	A	A
**2 weeks**	+	+	+	+	A	A	A	A
**4 weeks**	+	+	+	+	A	A	A	A
**H_2O_2 [mM]**								
**0.0**	+	NA	+	NA	A	NA	A	NA
**0.3**	±	NA	±	NA	A	NA	A	NA
**0.6**	±	NA	±	NA	A	NA	A	NA
**1.2**	±	NA	±	NA	A	NA	A	NA
**2.4**	±	NA	±	NA	A	NA	A	NA
**10.0**	X	NA	X	NA	X	NA	X	NA
**NaCl [M]**								
**0.0**	+	NA	+	NA	A	NA	A	NA
**0.4**	+	NA	++	NA	A(YY)	NA	A(YY)	NA
**0.8**	+	NA	++	NA	A(YY)	NA	A(YY)	NA
**1.6**	+	NA	++	NA	A(YY)	NA	A(YY)	NA

Supplementary Table 3. Genome assembly statistics for *Rm* ATCC 52813 and comparative organisms from the Mucoromycotina subphylum.

	Rhizopus microsporus **ATCC 52813**	***Rhizopus microsporus*** **ATCC 52814**	***Rhizopus microsporus*** **ATCC 11559**	***Rhizopus chinensis*** **CCTCCM201021**	***Rhizopus delemar*** **99-880**
Assembly length, bp	25,972,395	24,950,816	24,077,254	45,739,792	46,087,117
Number of scaffolds	131	560	595	3,281	81
Scaffold N50	8	8	87	463	6
Scaffold L50, bp	1,118,338	105,542	84,607	30,430	3,104,119
Number of contigs	823	649	653	3,374	389
Contig N50	111	84	96	469	46
Contig L50, bp	69,382	92,626	73,149	30,211	303,660
% GC	37.48	37.42	37.25	36.92	35.6

Supplementary Table 4. Homology support and functional assignment statistics for *Rm* ATCC 52813 gene models.

	Rhizopus microsporus **ATCC 52813**		***Rhizopus chinensis*** **CCTCCM201021**		***Rhizopus delemar*** **99-880**	
Number of genes	10,905	100%	17,676	100%	17,467	100%
Hits to databases:						
KEGG	8,119	74%	13,686	77%	11,456	66%
KOG	7,601	70%	12,878	73%	9,028	52%
Swissprot	7,688	71%	12,964	73%	10,609	61%
nr_b_b	7,835	72%	15,983	90%	10,565	60%
HMMPfam	6,587	60%	11,056	63%	9,030	52%
Number of genes with complete CDS	10,229	94%	16,036	91%	17,412	100%
Proteins with transmembrane helices	1,863	17%	2,934	17%	2,133	12%
Proteins with signal peptide	1,294	12%	1,960	11%	1,546	9%

Supplementary Table 5. Reproductive proteins across fungi. Unless otherwise noted, *A. nidulans* nomenclature is used following Dyer and O'Gorman[1]; [a]*S. cerevisiae* nomenclature; [b]*P. blakesleeanus* nomenclature; [c]*U. maydis* nomenclature.

Protein	LocusID	NCBI accession	Role in reproduction	Reference
**CpcA**	AN3675	CBF75634	Amino acid sensing	2
**CpcB**	AN4163	CBF74576	Amino acid sensing	2
**ImeB**	AN6243	CBF69873	Light response, sexual development, secondary metabolism	3
**LsdA**	AN2330	CBF86620	Osmotic stress sensing	4
**LreA**	AN3435	CBF82714	Blue light sensing	5
**LreB**	AN3607	CBF75789	Blue light sensing	5
**CryA**	AN0387	CBF89585	Blue light and UV sensing	6
**FphA**	AN9008	CBF84471	Red light sensing	7
**VeA/VosA**	AN1052/ AN1959	CBF88249/ CBF85898	Light/dark response regulator, secondary metabolite production	8,9
**VelB**	AN0363	CBF89638	Light/dark response regulator, secondary metabolite production	8
**LaeA**	AN0807	CBF88745	Light/dark response regulator, secondary metabolite production	8
**VelC**	AN2059	CBF86097	Light/dark response regulator, secondary metabolite production	10
**Pho80**	AN5156	CBF80977	Phosphorous sensing cyclin	11
**PhoA**	AN8261	CBF74234	Phosphorous sensing, interacts with Pho80	12
**FhbA**	AN7169	CBF78937	Nitrogen metabolism	13
**MAT1**	AN2755	CBF84081	Transcription factor: regulator of sexual reproduction	14
**MAT2**	AN4734	CBF76901	HMG box transcription factor: regulator of sexual reproduction	14
**SexP**[b]	NA	ABX27912	HMG box transcription factor: regulator of sexual reproduction	15
**bE1**[c]	UM00577	XP_011386823	Homeobox domain transcription factor: regulator of sexual reproduction	16
**bW1**[c]	UM00578	XP_011386405	Homeobox domain transcription factor: regulator of sexual reproduction	16
**Ram1p**[a]	YDL090C	NP_010193	Pheromone biogenesis: a-factor prenylation	17
**Ste24p**[a]	YJR117W	NP_012651	Pheromone biogenesis: a-factor proteolysis	18
**Ste14p**[a]	YDR410C	NP_010698	Pheromone biogenesis: a-factor carboxyl methylation	19
**Ste23p**[a]	YLR389C	NP_013493	Pheromone biogenesis: a-factor proteolysis	20
**Axl1p**[a]	YPR122W	NP_015447	Pheromone biogenesis: a-factor proteolysis	20
**Ste6p**[a]	YKL209C	NP_012713	Pheromone biogenesis: a-factor transporter	21
**Ste13p**[a]	YOR219C	NP_014862	Pheromone biogenesis: α-factor processing	22
**PpgA**	AN5791	CBF81183	Pheromone precursor (α type)	14
**PreA/GprB**	AN7743	CBF80019	GPCR: a type pheromone receptor	23,24
**PreB/GprA**	AN2520	CBF87016	GPCR: α type pheromone receptor	23,24
**GprD**	AN3387	CBF82810	GPCR	25
**GprK**	AN7795	CBF80123	GPCR	26
**TAR1**	NA	To be provided upon publication	GPCR: candidate risporic acid receptor	This study

**TAR2**	NA	To be provided upon publication	GPCR: candidate trisporic acid receptor	This study
**TAR3**	NA	To be provided upon publication	GPCR: candidate trisporic acid receptor	This study
**Gpa3**[c]	UM04474	P87034	*U. maydis* G protein α subunit: signaling to pheromone MAP kinase and cAMP cascades	27
**FadA**	AN0651	CBF89057	G protein α subunit: signaling to pheromone MAP kinase cascade	28
**SfaD**	AN0081	CBF90238	G protein β subunit: signaling to pheromone MAP kinase cascade	28
**GpgA**	AN2742	CBF84107	G protein γ subunit: signaling to pheromone MAP kinase cascade	29
**PhnA**	AN0082	CBF90234	Regulator of GpgA, SfaD and FlbA	30
**FlbA**	AN5893	CBF70620	Regulator of G-protein signaling	31
**Sql2**[c]	UM01516	XP_011387982	Regulator of Ras2	32
**RasA**	AN0182	CBF90024	Ras signaling protein involved in asexual development	2
**Ras2**[c]	UM01643	XP_011387629	Ras signaling protein involved in reproductive development, cAMP regulation, and filamentous growth	33
**Msn5p**[a]	YDR335W	NP_010622	Nuclear import and export of sex-related transcription factors	34
**Uac1**[c]	UM05232	XP_761379	Adenylate cyclase	35
**Uka1-1/1-2**[c]	UM01124	XP_757271	cAMP dependent protein kinase catalytic subunit	36
**Ubc1**[c]	UM06450	XP_762597	cAMP dependent protein kinase regulatory subunit	35
**Far1p**[a]	YJL157C	NP_012378	Pheromone regulation	37
**Cdc42p**[a]	YLR229C	NP_013330	Rho-GTPase, cell polarity	38
**Cdc24p**[a]	YAL041W	NP_009359	Guanyl nucleotide exchange factor for Cdc42, cell polarity	39
**Cdc28p**[a]	YBR160W	NP_009718	Cyclin-dependent kinase catalytic subunit	40
**Ste20p**[a]	YHL007C	NP_011856	Pheromone response	41
**SteD**	AN7252	CBF78763	Pheromone MAP kinase cascade regulator	14
**Ste5p**[a]	YDR103W	NP_010388	Pheromone MAP kinase cascade scaffold	42
**Ste20**	AN5674	CBF81427	MAP kinase, kinase, kinase, kinase (pheromone cascade)	1
**SteC**	AN2269	CBF86503	MAP kinase, kinase, kinase (pheromone cascade)	43
**Ste7**	AN3422	CBF82742	MAP kinase, kinase (pheromone cascade)	14
**MpkB**	AN3719	CBF75537	Map kinase (pheromone cascade)	14
**CandA-C**	AN2458	CBF86897	Regulate reproductive mode in response to light	44
**CandA-N**	AN10306	CBF86906	Regulate reproductive mode in response to light	44
**Dig1p**[a]	YPL049C	NP_015276	Regulation of mating-specific genes	45
**Dig2p**[a]	YDR480W	NP_010768	Regulation of mating-specific genes	45
**Msg5p**[a]	YNL053W	NP_014345	Regulation of pheromone MAP kinase cascade	46
**CsnA**	AN1491	CBF84973	Regulation of sexual development	47
**CsnB**	AN4783	CBF76790	Regulation of sexual development	47
**CsnD**	AN1539	CBF85069	Regulation of sexual development	47
**CsnE**	AN2129	CBF86237	Regulation of sexual development	47

**CsnG/AcoB**	AN3623	CBF75752	Regulation of sexual development	47
**StrA**	AN8071	CBF73824	Sexual and asexual development: striatin scaffolding and Ca signaling	48
**EsdC**	AN9121	CBF82506	Sexual development	49
**RcoA**	AN6505	CBF70872	Sexual vs asexual development, secondary metabolism	50
**PpoA/PpoC**	AN1967/ AN5028	CBF85912/ CBF76249	Sexual vs asexual development, secondary metabolism (oxylipin)	51
**PpoB**	AN6320	CBF69709	Sexual vs asexual development, secondary metabolism (oxylipin)	51
**Sir3p**[a]	YLR442C	NP_013547	Silencing MAT loci	52
**Sir4p**[a]	YDR227W	NP_010513	Silencing MAT loci	53
**HogA**	AN1017	CBF88327	Stress response	54
**AtfA**	AN2911	CBF83765	Stress response	55
**MsnA**	AN1652	CBF85299	Transcription factor	1
**StuA**	AN5836	CBF70741	Transcription factor	56
**DopA**	AN2094	CBF86166	Transcription factor	57
**MedA**	AN6230	CBF69898	Transcription factor	58
**FhpA**	AN4521	CBF77337	Transcription factor	59
**DevR**	AN7553	CBF79619	Transcription factor	60
**NosA**	AN1848	CBF85681	Transcription factor	61
**FlbE**	AN0721	CBF88918	Transcription factor	62
**FlbC**	AN2421	CBF86815	Transcription factor	63
**NsdC**	AN4263	CBF74364	Transcription factor	64
**NsdD**	AN3152	CBF83303	Transcription factor	31
**RosA**	AN5170	CBF81007	Transcription factor	65
**SteA**	AN2290	CBF86542	Transcription factor activated by pheromone MAP kinase cascade	66
**SilG**	AN0709	CBF88942	Transcription factor: light response	1
**SilA**	AN1893	CBF85772	Transcription factor: light response	1
**TrxA**	AN0170	CBF90049	Redox regulation	67
**NoxA**	AN5457	CBF81876	Redox regulation	68
**SidC**	AN0607	CBF89140	Iron regulation (NRPS)	69
**HxtA**	AN6923	CBF71745	Hexose transport	70
**MutA**	AN7349	CBF78568	Carbohydrate catabolism, mutanase activity	71
**SamB**	AN0078	CBF90245	Cell polarity	72
**GrrA**	AN10516	CBF74496	Meiotic structure development	73
**TubB**	AN0316	CBF89739	Microtubule assembly	74

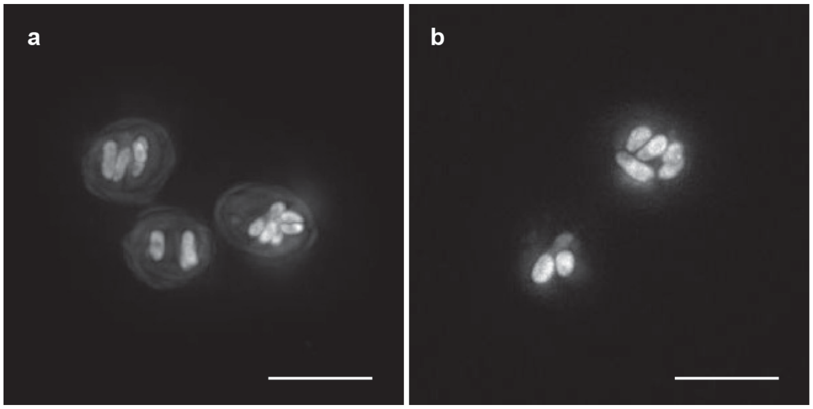

Supplementary Figure 1. *Burkholderia* endobacteria in sporangiospores of *Rm*. (a) ATCC 52813. **(b)** ATCC 52814. For visualization, bacteria expressed mCherry fluorescent protein in ATCC 52813 and YFP in ATCC 52814. Scale bars, 5 μm.

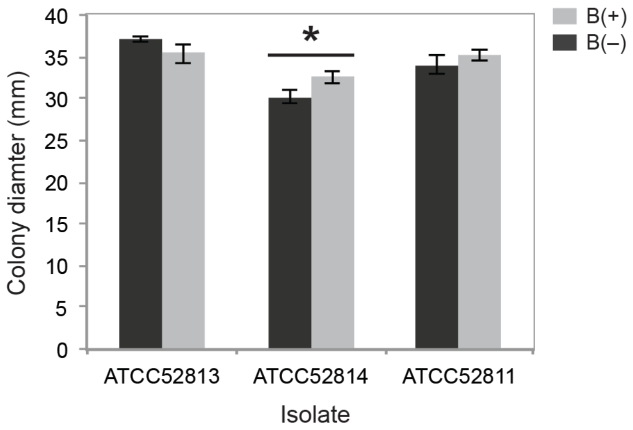

Supplementary Figure 2. Impact of *Burkholderia* endobacteria on *Rm* colony expansion. Colony diameter was measured after one day of growth. *, *t* test $0.05 \geq P > 0.01$. Error bars represent s.e.m.

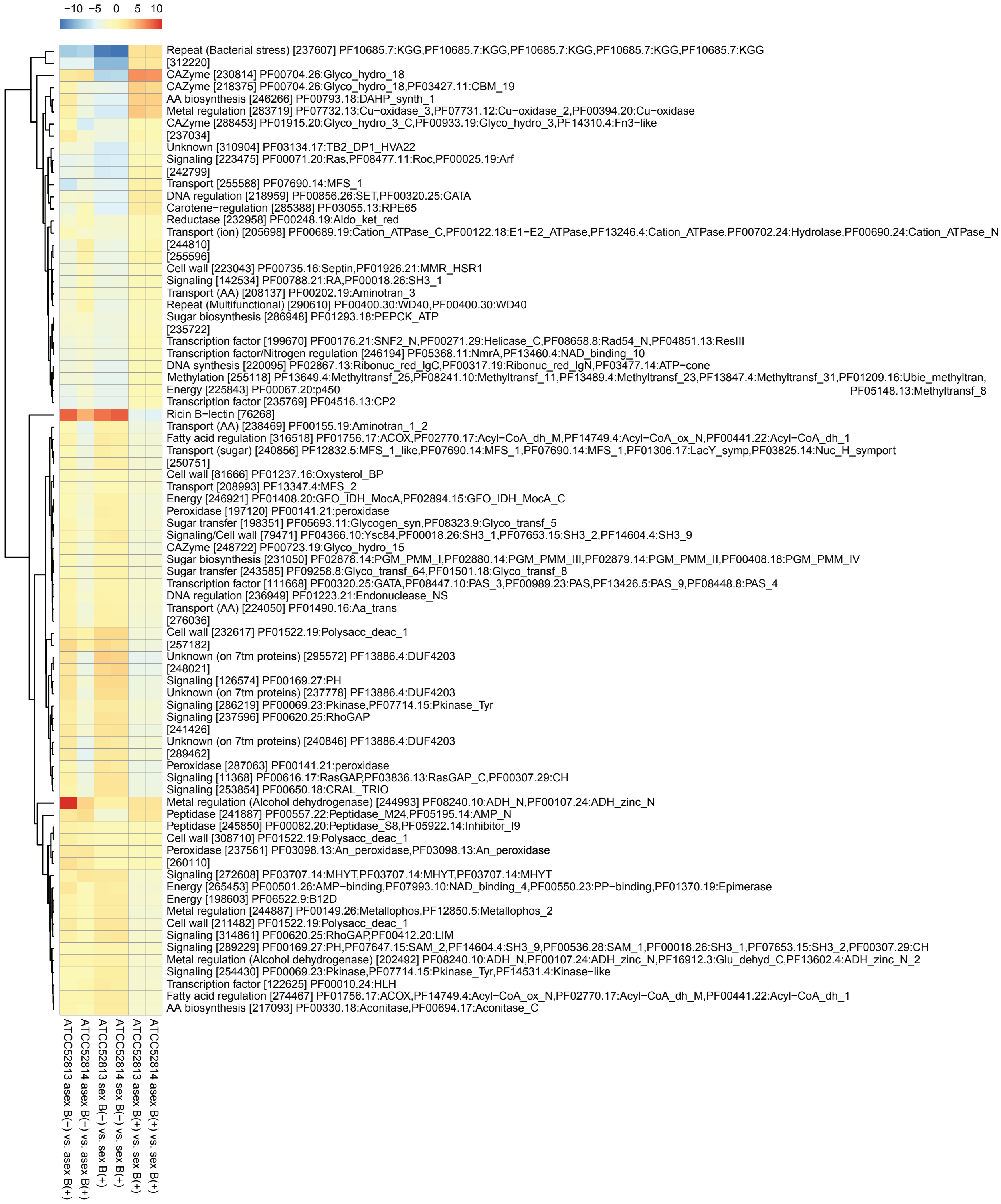

Supplementary Figure 3. Log2 fold change in expression of *Rm* genes displaying significant differences across all experimental comparisons. Broad categories (Signaling, Cell wall, etc) as well as all Pfam annotations are shown for each gene. *Rm* ATCC 52813 Protein IDs are shown in brackets.

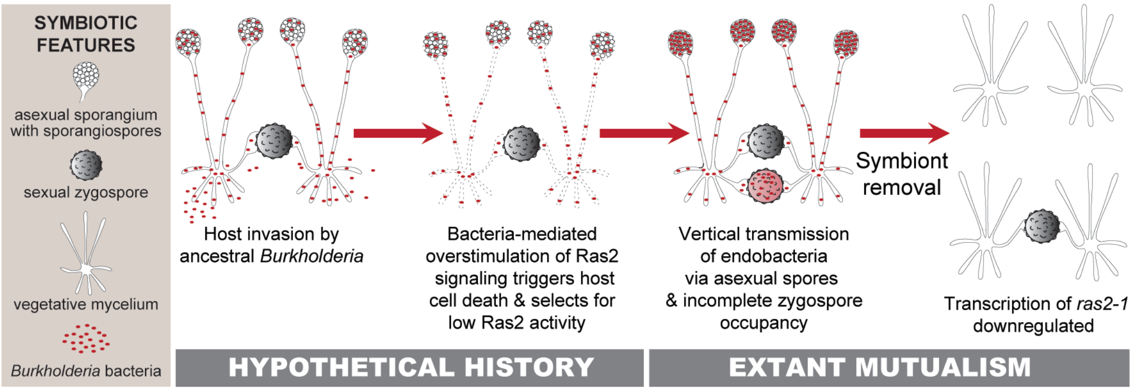

Supplementary Figure 4. Hypothetical evolutionary trajectory leading to the present-day *Rm-Burkholderia* mutualism.

Supplementary References

1 Dyer, P. S. & O'Gorman, C. M. Sexual development and cryptic sexuality in fungi: insights from *Aspergillus* species. *FEMS Microbiol. Rev.* **36**, 165-192, doi:10.1111/j.1574-6976.2011.00308.x (2012).

2 Hoffmann, B., Wanke, C., LaPaglia, S. K. & Braus, G. H. c-Jun and RACK1 homologues regulate a control point for sexual development in *Aspergillus nidulans*. *Mol. Microbiol.* **37**, 28-41, doi:10.1046/j.1365-2958.2000.01954.x (2000).

3 Bayram, Ö., Sari, F., Braus, G. H. & Irniger, S. The protein kinase ImeB is required for light-mediated inhibition of sexual development and for mycotoxin production in *Aspergillus nidulans*. *Mol. Microbiol.* **71**, 1278-1295, doi:10.1111/j.1365-2958.2009.06606.x (2009).

4 Lee, D. W. *et al.* The *lsdA* gene is necessary for sexual development inhibition by a salt in *Aspergillus nidulans*. *Curr. Genet.* **39**, 237-243 (2001).

5 Purschwitz, J. *et al.* Functional and physical interaction of blue- and red-light sensors in *Aspergillus nidulans*. *Curr. Biol.* **18**, 255-259, doi:10.1016/j.cub.2008.01.061 (2008).

6 Bayram, Ö., Biesemann, C., Krappmann, S., Galland, P. & Braus, G. H. More than a repair enzyme: *Aspergillus nidulans* photolyase-like CryA is a regulator of sexual development. *Mol. Biol. Cell* **19**, 3254-3262, doi:10.1091/mbc.E08-01-0061 (2008).

7 Blumenstein, A. *et al.* The *Aspergillus nidulans* phytochrome FphA represses sexual development in red light. *Curr. Biol.* **15**, 1833-1838, doi:10.1016/j.cub.2005.08.061 (2005).

8 Bayram, Ö. *et al.* VelB/VeA/LaeA complex coordinates light signal with fungal development and secondary metabolism. *Science* **320**, 1504–1506 (2008).

9 Ni, M. & Yu, J. H. A novel regulator couples sporogenesis and trehalose biogenesis in *Aspergillus nidulans*. *PLoS One* **2**, e970, doi:10.1371/journal.pone.0000970 (2007).

10 Park, H.-S. & Yu, J.-H. Genetic control of asexual sporulation in filamentous fungi. *Curr. Opin. Microbiol.* **15**, 669-677, doi:http://dx.doi.org/10.1016/j.mib.2012.09.006 (2012).

11 Wu, D. L., Dou, X. W., Hashmi, S. B. & Osmani, S. A. The Pho80-like cyclin of *Aspergillus nidulans* regulates development independently of its role in phosphate acquisition. *J. Biol. Chem.* **279**, 37693-37703, doi:10.1074/jbc.M403853200 (2004).

12 Bussink, H. J. & Osmani, S. A. A cyclin-dependent kinase family member (PHOA) is required to link developmental fate to environmental conditions in *Aspergillus nidulans*. *EMBO J.* **17**, 3990-4003, doi:10.1093/emboj/17.14.3990 (1998).

13 Baidya, S., Cary, J. W., Grayburn, W. S. & Calvo, A. M. Role of nitric oxide and flavohemoglobin homolog genes in *Aspergillus nidulans* sexual development and mycotoxin production. *Appl. Environ. Microb.* **77**, 5524-5528, doi:10.1128/aem.00638-11 (2011).

14 Paoletti, M. *et al.* Mating type and the genetic basis of self-fertility in the model fungus *Aspergillus nidulans*. *Curr. Biol.* **17**, 1384-1389 (2007).

15 Idnurm, A., Walton, F. J., Floyd, A. & Heitman, J. Identification of the sex genes in an early diverged fungus. *Nature* **451**, 193-196 (2008).

16 Kämper, J., Reichmann, M., Romeis, T., Bölker, M. & Kahmann, R. Multiallelic recognition: Nonself-dependent dimerization of the bE and bW homeodomain proteins in *Ustilago maydis*. *Cell* **81**, 73-83, doi:10.1016/0092-8674(95)90372-0 (1995).

17 Trueblood, C. E., Boyartchuk, V. L. & Rine, J. Substrate specificity determinants in the farnesyltransferase beta-subunit. *P. Natl. Acad. Sci. USA* **94**, 10774-10779, doi:10.1073/pnas.94.20.10774 (1997).

18 Fujimura-Kamada, K., Nouvet, F. J. & Michaelis, S. A novel membrane-associated metalloprotease, Ste24p, is required for the first step of NH_2-terminal processing of the yeast a-factor precursor. *J. Cell Biol.* **136**, 271-285, doi:10.1083/jcb.136.2.271 (1997).

19 Sapperstein, S., Berkower, C. & Michaelis, S. Nucleotide sequence of the yeast *STE14* gene, which encodes farnesylcysteine carboxyl methyltransferase, and demonstration of its essential role in a-factor export. *Mol. Cell. Biol.* **14**, 1438-1449 (1994).

20 Adames, N., Blundell, K., Ashby, M. N. & Boone, C. Role of yeast insulin-degrading enzyme homologs in propheromone processing and bud site selection. *Science* **270**, 464-467, doi:10.1126/science.270.5235.464 (1995).

21 McGrath, J. P. & Varshavsky, A. The yeast STE6 gene encodes a homolog of the mammalian multidrug resistance P-glycoprotein. *Nature* **340**, 400-404, doi:10.1038/340400a0 (1989).

22 Julius, D., Blair, L., Brake, A., Sprague, G. & Thorner, J. Yeast α factor is processed from a larger precursor polypeptide: The essential role of a membrane-bound dipeptidyl aminopeptidase. *Cell* **32**, 839-852, doi:10.1016/0092-8674(83)90070-3 (1983).

23 Dyer, P. S., Paoletti, M. & Archer, D. B. Genomics reveals sexual secrets of *Aspergillus*. *Microbiology* **149**, 2301–2303 (2003).

24 Seo, J. A., Han, K. H. & Yu, J. H. The gprA and gprB genes encode putative G protein-coupled receptors required for self-fertilization in *Aspergillus nidulans*. *Mol. Microbiol.* **53**, 1611-1623, doi:10.1111/j.1365-2958.2004.04232.x (2004).

25 Han, K. H., Seo, J. A. & Yu, J. H. A putative G protein-coupled receptor negatively controls sexual development in *Aspergillus nidulans*. *Mol. Microbiol.* **51**, 1333-1345, doi:10.1111/j.1365-2958.2003.03940.x (2004).

26 Wang, Y. *et al.* Characterizations and functions of regulator of G protein signaling (RGS) in fungi. *Appl Microbiol Biotechnol* **97**, 7977-7987, doi:10.1007/s00253-013-5133-1 (2013).

27 Regenfelder, E. *et al.* G proteins in *Ustilago maydis*: Transmission of multiple signals? *EMBO J.* **16**, 1934-1942, doi:10.1093/emboj/16.8.1934 (1997).

28 Rosén, S., Yu, J. H. & Adams, T. H. The *Aspergillus nidulans sfaD* gene encodes a G protein β subunit that is required for normal growth and repression of sporulation. *EMBO J.* **18**, 5592-5600, doi:10.1093/emboj/18.20.5592 (1999).

29 Seo, J. A., Han, K. H. & Yu, J. H. Multiple roles of a heterotrimeric G-protein γ-subunit in governing growth and development of *Aspergillus nidulans*. *Genetics* **171**, 81-89, doi:10.1534/genetics.105.042796 (2005).

30 Seo, J. A. & Yu, J. H. The phosducin-like protein PhnA is required for G βγ-mediated signaling for vegetative growth, developmental control, and toxin biosynthesis in *Aspergillus nidulans*. *Eukaryot. Cell* **5**, 400-410, doi:10.1128/ec.5.2.400-410.2006 (2006).

31 Han, K. H. *et al.* The *nsdD* gene encodes a putative GATA-type transcription factor necessary for sexual development of *Aspergillus nidulans*. *Mol. Microbiol.* **41**, 299-309, doi:10.1046/j.1365-2958.2001.02472.x (2001).

32 Müller, P., Katzenberger, J. D., Loubradou, G. & Kahmann, R. Guanyl nucleotide exchange factor Sql2 and Ras2 regulate filamentous growth in *Ustilago maydis*. *Eukaryot. Cell* **2**, 609-617, doi:10.1128/ec.2.3.609-617.2003 (2003).

33 Lee, N. & Kronstad, J. W. *ras2* controls morphogenesis, pheromone response, and pathogenicity in the fungal pathogen *Ustilago maydis*. *Eukaryot. Cell* **1**, 954-966, doi:10.1128/ec.1.6.954-966.2002 (2002).

34 Alepuz, P. M., Matheos, D., Cunningham, K. W. & Estruch, F. The *Saccharomyces cerevisiae* RanGTP-binding protein Msn5p is involved in different signal transduction pathways. *Genetics* **153**, 1219-1231 (1999).

35 Gold, S., Duncan, G., Barrett, K. & Kronstad, J. cAMP regulates morphogenesis in the fungal pathogen *Ustilago maydis*. *Gene. Dev.* **8**, 2805-2816, doi:10.1101/gad.8.23.2805 (1994).

36 Durrenberger, F., Wong, K. & Kronstad, J. W. Identification of a cAMP-dependent protein kinase catalytic subunit required for virulence and morphogenesis in *Ustilago maydis*. *P. Natl. Acad. Sci. USA* **95**, 5684-5689, doi:10.1073/pnas.95.10.5684 (1998).

37 Chenevert, J., Valtz, N. & Herskowitz, I. Identification of genes required for normal pheromone-induced cell polarization in *Saccharomyces cerevisiae*. *Genetics* **136**, 1287-1296 (1994).

38 Johnson, D. I. & Pringle, J. R. Molecular characterization of *CDC42*, a *Saccharomyces cerevisiae* gene involved in the development of cell polarity. *J. Cell Biol.* **111**, 143-152, doi:10.1083/jcb.111.1.143 (1990).

39 Ohya, Y., Miyamoto, S., Ohsumi, Y. & Anraku, Y. Calcium-sensitive *cls4* mutant of *Saccharomyces cerevisiae* with a defect in bud formation. *J. Bacteriol.* **165**, 28-33 (1986).

40 Lörincz, A. T. & Reed, S. I. Primary structure homology between the product of yeast cell division control gene *CDC28* and vertebrate oncogenes. *Nature* **307**, 183-185, doi:10.1038/307183a0 (1984).

41 Leberer, E., Dignard, D., Harcus, D., Thomas, D. Y. & Whiteway, M. The protein kinase homolog Ste20p is required to link the yeast pheromone response G-protein βγ subunits to downstream signaling components. *EMBO J.* **11**, 4815-4824 (1992).

42 Printen, J. A. & Sprague, G. F. Protein-protein interactions in the yeast pheromone response pathway: Ste5p interacts with all members of the MAP kinase cascade. *Genetics* **138**, 609-619 (1994).

43 Wei, H. J., Requena, N. & Fischer, R. The MAPKK kinase SteC regulates conidiophore morphology and is essential for heterokaryon formation and sexual development in the homothallic fungus *Aspergillus nidulans*. *Mol. Microbiol.* **47**, 1577-1588, doi:10.1046/j.1365-2958.2003.03405.x (2003).

44 Helmstaedt, K. *et al.* Recruitment of the inhibitor Cand1 to the cullin substrate adaptor site mediates interaction to the neddylation site. *Mol. Biol. Cell* **22**, 153-164, doi:10.1091/mbc.E10-08-0732 (2011).

45 Tedford, K., Kim, S., Sa, D., Stevens, K. & Tyers, M. Regulation of the mating pheromone and invasive growth responses in yeast by two MAP kinase substrates. *Curr. Biol.* **7**, 228-238, doi:10.1016/s0960-9822(06)00118-7 (1997).

46 Doi, K. *et al.* MSG5, a novel protein phosphatase promotes adaptation to pheromone response in *Saccharomyces cerevisiae*. *EMBO J.* **13**, 61-70 (1994).

47 Busch, S. *et al.* An eight-subunit COP9 signalosome with an intact JAMM motif is required for fungal fruit body formation. *P. Natl. Acad. Sci. USA* **104**, 8089-8094, doi:10.1073/pnas.0702108104 (2007).

48 Wang, C. L., Shim, W. B. & Shaw, B. D. *Aspergillus nidulans* striatin (StrA) mediates sexual development and localizes to the endoplasmic reticulum. *Fungal Genet. Biol.* **47**, 789-799, doi:10.1016/j.fgb.2010.06.007 (2010).

49 Han, K. H. *et al.* The *Aspergillus nidulans esdC* (early sexual development) gene is necessary for sexual development and is controlled by *veA* and a heterotrimeric G protein. *Fungal Genet. Biol.* **45**, 310-318, doi:10.1016/j.fgb.2007.09.008 (2008).

50 Todd, R. B., Hynes, M. J. & Andrianopoulos, A. The *Aspergillus nidulans rcoA* gene is required for *veA*-dependent sexual development. *Genetics* **174**, 1685-1688, doi:10.1534/genetics.106.062893 (2006).

51 Tsitsigiannis, D. I., Kowieski, T. M., Zarnowski, R. & Keller, N. P. Three putative oxylipin biosynthetic genes integrate sexual and asexual development in *Aspergillus nidulans*. *Microbiology* **151**, 1809-1821, doi:10.1099/mic.0.27880-0 (2005).

52 Shore, D., Squire, M. & Nasmyth, K. A. Characterization of two genes required for the position-effect control of yeast mating-type genes. *EMBO J.* **3**, 2817-2823 (1984).

53 Ivy, J. M., Klar, A. J. S. & Hicks, J. B. Cloning and characterization of 4 SIR genes of *Saccharomyces cerevisiae*. *Mol. Cell. Biol.* **6**, 688-702 (1986).

54 Kawasaki, L., Sanchez, O., Shiozaki, K. & Aguirre, J. SakA MAP kinase is involved in stress signal transduction, sexual development and spore viability in *Aspergillus nidulans*. *Mol. Microbiol.* **45**, 1153-1163, doi:10.1046/j.1365-2958.2002.03087.x (2002).

55 Lara-Rojas, F., Sánchez, O., Kawasaki, L. & Aguirre, J. *Aspergillus nidulans* transcription factor AtfA interacts with the MAPK SakA to regulate general stress responses, development and spore functions. *Mol. Microbiol.* **80**, 436-454, doi:10.1111/j.1365-2958.2011.07581.x (2011).

56 Wu, J. G. & Miller, B. L. *Aspergillus* asexual reproduction and sexual reproduction are differentially affected by transcriptional and translational mechanisms regulating stunted gene expression. *Mol. Cell. Biol.* **17**, 6191-6201 (1997).

57 Pascon, R. C. & Miller, B. L. Morphogenesis in *Aspergillus nidulans* requires Dopey (DopA), a member of a novel family of leucine zipper-like proteins conserved from yeast to humans. *Mol. Microbiol.* **36**, 1250-1264, doi:10.1046/j.1365-2958.2000.01950.x (2000).

58 Busby, T. M., Miller, K. Y. & Miller, B. L. Suppression and enhancement of the *Aspergillus nidulans medusa* mutation by altered dosage of the *bristle* and *stunted* genes. *Genetics* **143**, 155-163 (1996).

59 Lee, B. Y. *et al.* Screening of growth- or development-related genes by using genomic library with inducible promoter in *Aspergillus nidulans*. *J. Microbiol.* **43**, 523-528 (2005).

60 Tüncher, A., Reinke, H., Martic, G., Caruso, M. L. & Brakhage, A. A. A basic-region helix-loop-helix protein-encoding gene (*devR*) involved in the development of *Aspergillus nidulans*. *Mol. Microbiol.* **52**, 227-241, doi:10.1111/j.1365-2958.2003.03961.x (2004).

61 Vienken, K. & Fischer, R. The Zn(II)$_2$Cys$_6$ putative transcription factor NosA controls fruiting body formation in *Aspergillus nidulans*. *Mol. Microbiol.* **61**, 544-554, doi:10.1111/j.1365-2958.2006.05257.x (2006).
62 Kwon, N. J., Shin, K. S. & Yu, J. H. Characterization of the developmental regulator FlbE in *Aspergillus fumigatus* and *Aspergillus nidulans*. *Fungal Genet. Biol.* **47**, 981-993, doi:10.1016/j.fgb.2010.08.009 (2010).
63 Kwon, N. J., Garzia, A., Espeso, E. A., Ugalde, U. & Yu, J. H. FlbC is a putative nuclear C_2H_2 transcription factor regulating development in *Aspergillus nidulans*. *Mol. Microbiol.* **77**, 1203-1219, doi:10.1111/j.1365-2958.2010.07282.x (2010).
64 Kim, H. R., Chae, K. S., Han, K. H. & Han, D. M. The *nsdC* gene encoding a putative C_2H_2-type transcription factor is a key activator of sexual development in *Aspergillus nidulans*. *Genetics* **182**, 771-783, doi:10.1534/genetics.109.101667 (2009).
65 Vienken, K., Scherer, M. & Fischer, R. The Zn(II)$_2$Cys$_6$ putative *Aspergillus nidulans* transcription factor repressor of sexual development inhibits sexual development under low-carbon conditions and in submersed culture. *Genetics* **169**, 619-630, doi:10.1534/genetics.104.030767 (2005).
66 Vallim, M. A., Miller, K. Y. & Miller, B. L. *Aspergillus* SteA (Sterile12-like) is a homeodomain-C_2/H_2-Zn^{+2} finger transcription factor required for sexual reproduction. *Mol. Microbiol.* **36**, 290-301, doi:10.1046/j.1365-2958.2000.01874.x (2000).
67 Thön, M., Al-Abdallah, Q., Hortschansky, P. & Brakhage, A. A. The thioredoxin system of the filamentous fungus *Aspergillus nidulans*: Impact on development and oxidative stress response. *J. Biol. Chem.* **282**, 27259-27269, doi:10.1074/jbc.M704298200 (2007).
68 Lara-Ortíz, T., Riveros-Rosas, H. & Aguirre, J. Reactive oxygen species generated by microbial NADPH oxidase NoxA regulate sexual development in *Aspergillus nidulans*. *Mol. Microbiol.* **50**, 1241-1255, doi:10.1046/j.1365-2958.2003.03800.x (2003).
69 Eisendle, M. *et al.* The intracellular siderophore ferricrocin is involved in iron storage, oxidative-stress resistance, germination, and sexual development in *Aspergillus nidulans*. *Eukaryot. Cell* **5**, 1596-1603, doi:10.1128/ec.00057-06 (2006).
70 Wei, H. J. *et al.* A putative high affinity hexose transporter, hxtA, of *Aspergillus nidulans* is induced in vegetative hyphae upon starvation and in ascogenous hyphae during cleistothecium formation. *Fungal Genet. Biol.* **41**, 148-156, doi:10.1016/j.fgb.2003.10.006 (2004).
71 Wei, H. J., Scherer, M., Singh, A., Liese, R. & Fischer, R. Aspergillus nidulans alpha-1,3 glucanase (mutanase), mutA, is expressed during sexual development and mobilizes mutan. *Fungal Genet. Biol.* **34**, 217-227, doi:10.1006/fgbi.2001.1303 (2001).
72 Krüger, M. & Fischer, R. Integrity of a Zn finger-like domain in SamB is crucial for morphogenesis in ascomycetous fungi. *EMBO J.* **17**, 204-214, doi:10.1093/emboj/17.1.204 (1998).
73 Krappmann, S. *et al.* The *Aspergillus nidulans* F-box protein GrrA links SCF activity to meiosis. *Mol. Microbiol.* **61**, 76-88, doi:10.1111/j.1365-2958.2006.05215.x (2006).
74 Kirk, K. E. & Morris, R. N. The *tubB* α-tubulin gene is essential for sexual development in *Aspergillus nidulans*. *Gene. Dev.* **5**, 2014-2023 (1991).
