# Supplementary material for: Bacterial endosymbionts influence host sexuality and reveal reproductive genes of early divergent fungi
Source: Nat Commun. 2017 Nov 29;8:1843. doi: 10.1038/s41467-017-02052-8 (PMC5705715; doi:10.1038/s41467-017-02052-8)
Supplement: Supplementary file 4 — Description of Additional Supplementary Files [file 41467_2017_2052_MOESM4_ESM.pdf]

Description of Additional Supplementary Files

File Name: Supplementary Data 1
Description: RNAseq results.

File Name: Supplementary Data 2
Description: OrthoMCL gene clusters.

File Name: Supplementary Data 3
Description: GPCR sequence alignment.

File Name: Supplementary Data 4
Description: GPCR phylogeny.

File Name: Supplementary Data 5
Description: Ras sequence alignment.
